# Between acceptance and change – a qualitative study about how leaders deal with bureaucracy

**DOI:** 10.3389/fpsyg.2026.1739144

**Published:** 2026-05-12

**Authors:** Jule Uhl, Maren M. Michaelsen, Johanna Hertzberg, Mira Kriegesmann, Magdalena Wallkamm, Tobias Esch

**Affiliations:** Institute for Integrative Health Care and Health Promotion, Faculty of Health, Witten/Herdecke University, Witten, Germany

**Keywords:** behavior change resource model, bureaucracy, coping strategies, grounded theory, inner sense of safety, leadership, meaningful work, salutogenesis

## Abstract

**Introduction:**

Bureaucracy is characterized by narrowly defined rules and regulations. Such structures can limit both the performance of organizations and the health of employees.

**Materials and methods:**

Guided interviews were conducted with leaders from the German health, education and social service sectors who self-reported as motivated and healthy despite bureaucratic requirements. Transcriptions were iteratively analyzed using the grounded theory methodology. Quantitative surveys provide additional information.

**Results:**

The leaders (*n* = 16) reported diverse perspectives on bureaucracy, highlighting its causes, consequences and coping strategies. The effects of bureaucracy were predominantly described as negative. However, bureaucracy can also create an inner sense of safety. Resources and coping mechanisms relating to health, emotions, cognition, leadership, relationships, processes and society were identified, including different forms of acceptance and change initiatives.

**Conclusion:**

The experience of meaningfulness of bureaucratic regulations appears to be crucial for successfully managing bureaucratic demands. Bureaucracy offers protective effects, such as inner sense of safety and justice at the societal level. However, it is also associated with significant negative consequences, such as stress and restrictions in decision-making. The identified resources and coping mechanisms should be fostered in organizations to ensure health and motivation of leaders. Further research should explore whether certain bureaucratic regulations could be reduced, e.g., by strengthening inter-personal trust and increased inner sense of safety.

## 1 Introduction

Bureaucratic systems, inherently designed for stability and standardization (Weber, 1922), appear fundamentally at odds with the growing need for flexibility and agility in the modern working world (Bal and Izak, 2021). Flexibility requires adaptability and decentralized decision-making, characteristics that contradict the foundational principles of bureaucracy, which are among others hierarchical emphasis, tightly defined rules and regulations. Obstacles to agility in public sector organizations include the need for a broad foresight of budget utilization and political restrictions, as summarized by Shah and Stephens (2005). At the same time, in a constantly evolving working environment, the ability to respond flexibly to changes are necessary to remain competitive – whether it involves clients, members, employees, students or patients (Beilstein et al., 2021; Dias and Escoval, 2014; van Gool et al., 2022). This conflict can be a stressor for both employers and employees; potentially affecting the physical and mental well-being (stress reaction), depending on the individual demands and the resources of the person concerned (Esch, 2002). In particular, leaders face role conflicts: they must communicate bureaucratic requirements, reconcile conflicting interests across different hierarchical levels, and be accountable for their implementation (Birken et al., 2018; Franke et al., 2015). Beyond that, numerous bureaucratic requirements can severely restrict employees´ creative leeway (Hirst et al., 2011).

Workplace stressors, such as the conflict between bureaucracy and the demands of modern work environments, can lead to employee dissatisfaction, reduce motivation, and increase health problems such as burnout, musculoskeletal disorders and cardiovascular diseases (Gunderman and Lynch, 2018; Rothe et al., 2017). A meta-study reveals that job insecurity and high job demands are significantly linked to a higher risk of physical health problems ranging from self-reported poor health and medically diagnosed conditions, diminished mental well-being and increased mortality (Goh et al., 2015, 2016). These issues may contribute to higher turnover rates, threatening organizational stability and impeding its capacity to innovate. Rule and LeGouill (2019) emphasize that bureaucracy restricted medical research activities under the pretense of adhering to *good clinical practice*. They argue that stringent regulations demotivate scientists from investing their time and energy in research and development. Additionally, an excessive burden of bureaucratic tasks can increase the workload of medical staff, thereby compromising patient safety (Heck et al., 2021).

Since the 19th century, bureaucracy has been the dominant paradigm for leadership and management (Bush, 2019). Its key characteristics include a strong emphasis on hierarchy, vertical accountability, the division of labor based on expertise, narrowly defined rules and regulations, and impersonal relationships rooted in formal roles (Bush, 2019; Weber, 1922). Many work environments are heavily shaped by bureaucratic procedures and regulations, which may be either organization-specific (e.g., vacation request) or industry-specific (e.g., medical device certification) (DiMaggio and Powell, 1983). Bureaucracy translates legitimate political authority into practice and is thus considered as the foundation of society (Weber, 1978; cited in Kalucza and Hattke, 2020).

Efforts to reduce bureaucracy have long been promoted in Germany through various initiatives, such as in health care system (German Federal Ministry of Health, 2023), higher education (University forum, 2023) and the social sector (Lahrmann, 2011). In March 2024, the Federal Cabinet adopted the fourth *Bureaucracy Reduction Act* (BEG IV) as part of the *Meseberg Relief Package,* aiming to save over three billion euros annually. The bureaucracy cost index is anticipated to fall to an all-time low (German Federal Ministry of Justice, 2024a). BEG IV focuses on reducing reporting obligations, promoting digitization, and simplifying procedures (German Federal Ministry of Justice, 2024b).

Despite these political efforts, the potentially detrimental effects of bureaucracy or perceived overregulation on individuals, particularly leaders, have received limited attention in scientific literature.

Previous administrative and occupational research suggest that bureaucracy is generally perceived as detrimental to health and other outcomes, such as motivation and process-efficiency (Gunderman and Lynch, 2018; Johansson, 2016; Patel, 2013; Rule and LeGouill, 2019; Scott, 2021). Studies highlight the negative influences of bureaucracy on employees (e.g., Gunderman and Lynch, 2018; Johansson, 2016; Patel, 2013; Scott, 2021), although a considerable gap in research persist. Less is known about the coping strategies employees employ to maintain motivation and health. Successfully managing stress is essential for sustaining and promoting health in a globally interconnected, fast-changing world.

In order to fill this research gap, we examined how bureaucracy is perceived by leaders in the work environment within the health, education and social sectors in Germany. Leaders in an intermediary position between top management and operational level, in particular, face significant strain due to their intermediary role and the responsibility of balancing organizational demands with employees´ needs (Birken et al., 2018; Franke et al., 2015). Focusing on leaders provides a valuable lens, as leaders often play a crucial role in influencing employees through their leadership style (e.g., communication) (Othman et al., 2017) or as (un)conscious role models (e.g., by embodying their values) (Dietz et al., 2020). Furthermore, leaders are known to be key predictors of employee absenteeism within their department (Kuoppala et al., 2008; Løkke, 2022), making them vital multipliers of both health issues and health promotion within their organizations.

Our study introduces a novel resource-based perspective by examining how leaders navigate bureaucracy. This shift toward a resource-oriented approach is particularly relevant, especially given the growing importance of positive psychology. Two quantitative accompanying surveys collected additional information.

## 2 Materials and methods

The positive vote of this qualitative study was obtained from the Institutional Review Board of the University [Witten/Herdecke University] (no. S-247/2023).

### 2.1 Research objectives

The aim of this study was to determine: (1) how leaders characterize bureaucracy within their work environment, (2) its impact on their roles and well-being, and (3) the resources and coping mechanisms they perceive as essential for navigating bureaucratic contexts while maintaining both health and motivation.

### 2.2 Study design

The study adopted an exploratory qualitative approach, utilizing guideline-based expert interviews in conjunction with grounded theory methodology (Strauss and Corbin, 1998), followed by quantitative surveys. The design was used to facilitate both investigator-triangulation and method-triangulation. The entire research project was conducted in German.

The interviews were conducted one-on-one between November 2023 and February 2024 via video telephony. The interviews were transcribed verbatim. Codes were reconciled by authors. Each interview had a duration of approximately 45 min. Additionally, two quantitative surveys were administered (a) immediately following the interviews aimed gaining a better understanding of the sample (e.g., socio-demographic data) and (b) 2–5 months later, in April 2024.

**Figure 1:**
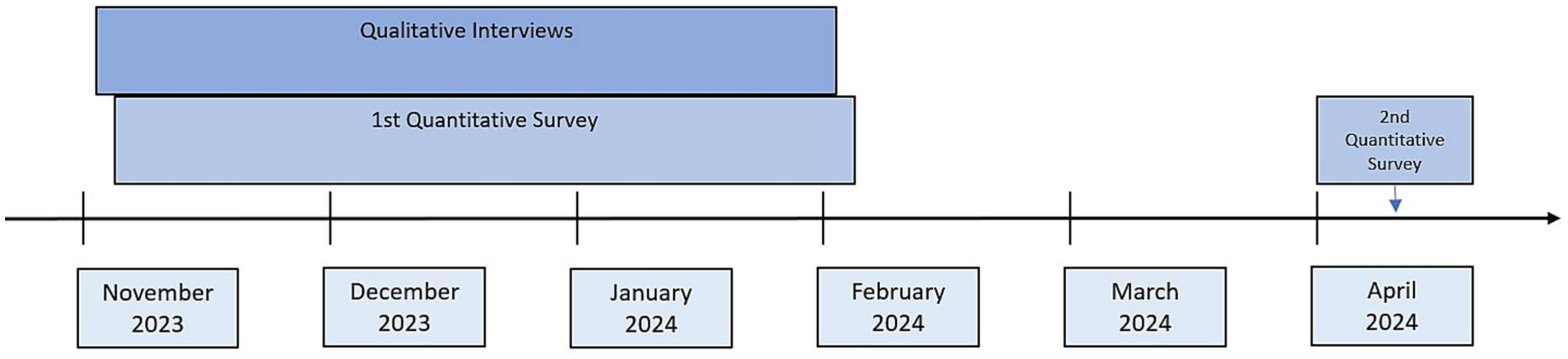
Timeline of data collection.

The timeline of the three study arms is shown in Figure 1.

### 2.3 Sample

#### 2.3.1 Recruitment

Interview partners were selected via personal contacts, online research, and recommendations from the target group (convenience sample), as well as referrals from other scientists and leaders (gatekeepers). A flyer was emailed to potential participants and gatekeepers. Case selection aimed for informational power, focusing on relevant theoretical concepts (Malterud et al., 2016).

#### 2.3.2 Inclusion and exclusion criteria

Participants were individuals with management responsibility (team or department leadership with budget oversight) in organizations within the health, education, or social services sectors in Germany. Eligibility required participants to perceive bureaucracy as integral part of their work and self-assess consider themselves both motivated and feeling healthy. While membership in their organization for more than five years was preferred, it was not mandatory. Exclusion criteria included individuals who felt significantly burdened by their work or had cognitive impairments. The assessment of the criteria was based on the participants´ self-evaluation.

Although all participants rated themselves as healthy, the interviews revealed variations in their satisfaction with their health (see *Health Satisfaction*).

### 2.4 Qualitative data collection, analysis and evaluation

Data collection and analysis followed grounded theory methodology as outlined by Strauss and Corbin (1998), aiming to empirically develop a middle-range theory (Glaser and Strauss, 1967; Kuckartz, 2018; Ruppel and Mey, 2015).

The interview guideline was developed in accordance with Helfferich (2011) and adhered to the stages of collection, verification, sorting, and subsumption. It was reviewed by an interdisciplinary analysis group consisting of three qualitative researchers external to the project. The guideline covered the following topics: (1) perception of bureaucracy, (2) bureaucracy and leadership, (3) consequences of bureaucracy, (4) personal health, (5) resources and coping strategies, (6) potential interventions or assistance, and (7) conclusion. Verbatim transcription was carried out following the methods outlined by Dresing and Pehl (2018). The managers were not provided with an *a priori* operationalization of bureaucracy so that participants could focus on those bureaucratic requirements that are most relevant to their respective work realities.

Due to the nature of the grounded theory approach, data collection and analysis were interdependent. Due to the high heterogeneity of the sample (health, social, education sectors), the modern approach of information power (Malterud et al., 2016) was chosen instead of the theoretical saturation (Corbin and Strauss, 2015) approach. Data analysis was guided by the research questions, therefore not all topics identified in the data were coded (Kuckartz, 2018).

**Figure 2:**
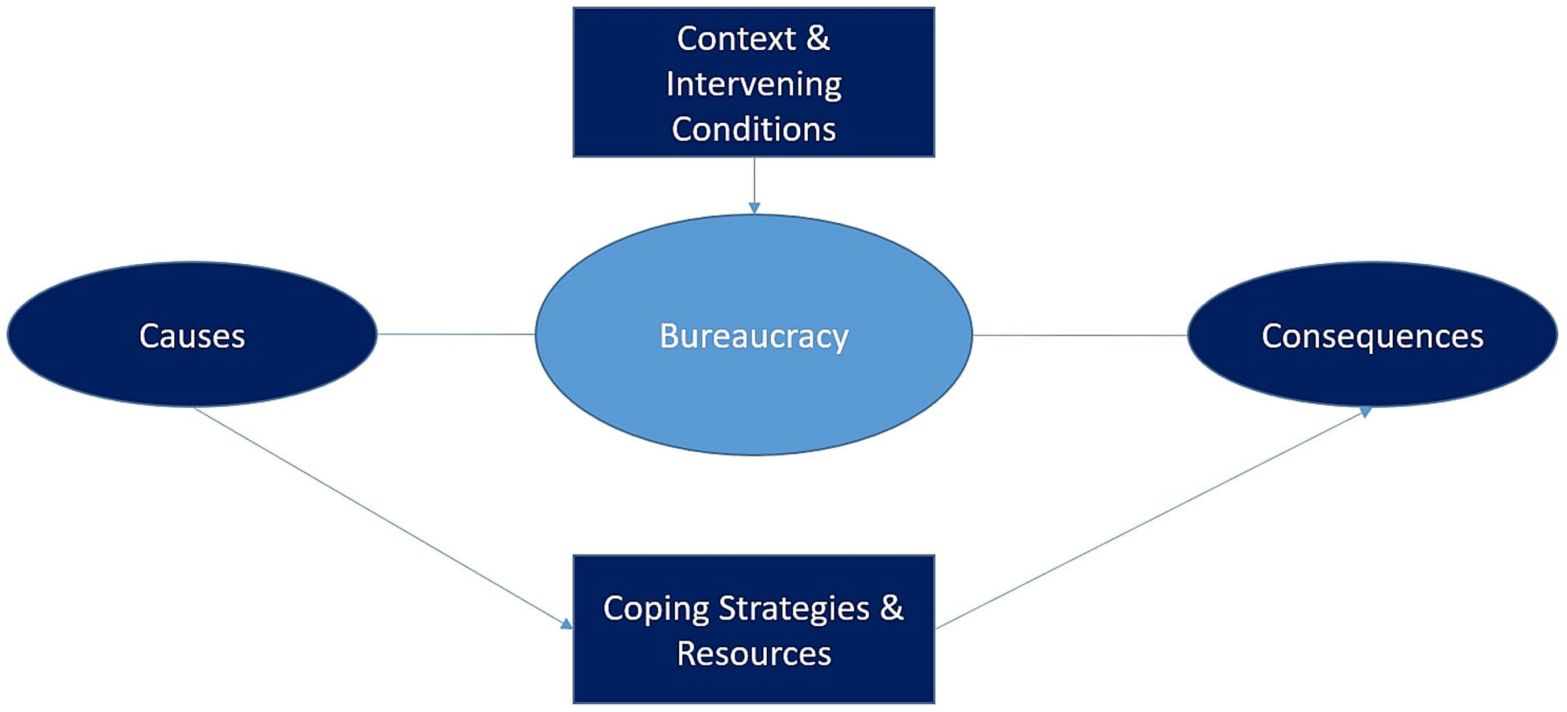
Axial coding model (Strauss and Corbin, 1998).

Following Strauss and Corbin (1998), coding occurred in three forms: *open coding*, *axial coding* (see Figure 2), and *selective coding*. Data analysis was performed using the *MAXQDA* program. Through a multi-stage process, codes were systematically developed from the material, adhering to the recommendation for as precise categories as possible (Kuckartz, 2018).

Investigator-triangulation ensured the robustness and validity of the findings by integrating all authors´ perspectives. Discrepancies in transcript interpretation were resolved through a reconciliation process involving cross-checking and consensus-building. Project staff contributed questions on interpretation and a final group discussion clarified ambiguities, resulting in a consensual summary for each interview.

The category system was generated based on this methodology (see Supplementary material 1).

### 2.5 Quantitative survey

Two accompanying surveys were carried out to gain deeper insight into how health and motivation can be maintained in a bureaucratic work environment. The quantitative surveys were conducted primarily to provide a better description of the sample. Immediately after completing the qualitative interviews (November 2023 – February 2024), a short, approximately five-minute quantitative follow-up survey was conducted via *LimeSurvey*. Two to five months after the interviews (April 2024), a second survey was carried out (see Figure 1).

#### 2.5.1 First quantitative survey

The first survey collected sociodemographic data, particularly: gender, year of birth, type of living (alone, with partner, with(out) children, flat share, other type of living), highest general school-leaving qualification, vocational training qualification. Additionally, information on the conditions of employment (contractual and actual working hours; open-ended response), extent of bureaucracy (percentages of working time for organization-specific, sector-specific, and other bureaucratic requirements; data preparation: grouped in 10 per cent increments), place of work (percentages of working time in the office, in the home office, and off-site), commuting time (open-ended response), and regular commuting (*on foot, by bike, by public transport, by car, in a carpool*), work experience in years (sector, organization, position), intention to leave jobs, (“Can you imagine leaving the organization for another job in the next 10 years?” Answer options: *yes*, *rather yes*, *rather no*, *no*, and *I will retire in the next 10 years*) were collected.

The results of the first quantitative survey were analyzed descriptively (see *Sample*).

#### 2.5.2 Second quantitative survey

To gain a deeper understanding and ensure methodological triangulation in the contextualization of the qualitative interview data, a quantitative follow-up surveys were conducted. In the spirit of member checking (Birt et al., 2016; Harvey, 2015; Lincoln and Guba, 1985), an additional follow-up survey was conducted, in which experts were asked, among other things, to evaluate the developed hypotheses. These data can, among other things, be helpful in the conception of a follow-up project in the context of a participatory project proposal. Two to five months after the interviews, the follow-up survey was sent out (April 2024, see Figure 1). The survey aimed to examine the key findings in greater depth and provide suggestions for further research questions. The survey was designed to take 5–10 min and was conducted via *LimeSurvey*.

The participants were asked at the beginning whether they situation remained the same as at the time of the interview (open-ended response).

Four items were developed to assess enduring of bureaucratic regulations and their potential impact on health and motivation. Respondents rated their agreement with statements on a five-point Likert scale (1 = *completely disagree* to 5 = *fully agree*). One example item is: “I accept bureaucratic requirements, even if I do not consider them meaningful.” Additionally, the assessment of uncertainty and error culture was surveyed through two developed items. One item stated: “Insecure employees benefit from bureaucratic regulations”.

The work-related *sense of coherence* (*SOC*) was measured using the Work-SoC scale (Bauer et al., 2015). Respondents were asked to rate how they generally perceived their current work situation using nine pairs of words, such as “structured” and “chaotic.” Seven response options lay between these poles (1 = least pronounced; e.g., *meaningless*; 7 = most pronounced, e.g., *meaningful*). The same nine word pairs were adapted in a second question set focused on typical bureaucratic requirements in the work context. The work-related *SOC* was derived from the three subscales of *comprehensibility* (manageable, structured, clear, predictable), *manageability* (controllable, steerable), and *meaningfulness* (meaningful, significant, worthwhile).

The Brief Sense of Community Scale (Wombacher et al., 2010) was used to operationalize the sense of belonging. Eight items each were rated on a five-point scale (1 = *does not apply at* all to 5 = *applies fully*) regarding agreement with the team, organization, and sector, e.g., “I feel like a part of this organization”.

The items were analyzed descriptively. Sum scores were calculated for the *SOC* and the *Community Scale*.

## 3 Results

### 3.1 Sample

Sixteen leaders participated in the study (I01-I16), all were interviewed, while 15 leaders took part in the first and 14 participants in the second quantitative survey. Sociodemographic data of the study participants are shown in Table 1.

**Table 1:** Sociodemographic data participant interviews.

Data source	Categories	Total *n* (%)
Interview partner (*n* = 16)	Gender	
	Female	10 (62.5%)
	Male	6 (37.5%)
	Age (grouped)	
	30 to 39 years	1 (6.25%)
	40 to 49 years	3 (18.75%)
	50 to 59 years	7 (43.75%)
	60 to 69 years	5 (31.25%)
First quantitative survey (*n* = 15)	Gender	
	Female	8 (53.33%)
	Male	6 (40.00%)
	*Missing data*	1 (6.67%)
	Age (grouped)	
	30 to 39 years	1 (6.67%)
	40 to 49 years	3 (20.00%)
	50 to 59 years	5 (33.33%)
	60 to 69 years	5 (33.33%)
	*Missing data*	1 (6.67%)
	Living situation	
	With partner and child(ren)	6 (40.00%)
	With partner without child(ren)	5 (33.33%)
	Alone	3 (20.00%)
	*Missing data*	1 (6.67%)
	Highest general education qualification	
	German (General or Specialized) University Entrance Qualification (Abitur)	14 (93.33%)
	*Missing data*	1 (6.67%)
	Highest vocational qualification	
	Master’s degree, diploma, state examination	8 (53.33%)
	Doctorate	4 (26.67%)
	Qualification from a technical, master, or engineering school, administration and business academy, or specialized academy	1 (6.67%)
	Vocational qualification with professional school-based training (vocational school, college school)	1 (6.67%)
	*Missing data*	1 (6,67%)
Second quantitative survey (*n* = 14)	Gender	
	Female	8 (57.14%)
	Male	6 (42.86%)
	Age (grouped)	
	30 to 39 years	1 (7.14%)
	40 to 49 years	3 (21.43%)
	50 to 59 years	6 (42.86%)
	60 to 69 years	3 (21.43%)
	Over 69 years	1 (7.14%)

#### 3.1.1 First quantitative survey

Fifteen participants (93.8% of the sample) took part in the first survey, whereby the majority of them were female (*n* = 8). In this survey, both the 50–59 and 60–69 age groups were equally represented (*n* = 5 per group). Living with a partner and child(ren) was the most commonly reported living arrangement (*n* = 6). All had a German university entrance qualification (Abitur) as their highest general education qualification (*n* = 14; *missing*: *n* = 1) and the majority had a Master’s degree, diploma or state examination as their highest vocational qualification (*n* = 8) (see Table 1).

The contractual working hours were mostly between 37.5 and 40 hours/week (*n* = 13). Two other individuals indicated that there were no fixed hours. The actual weekly working hours were between 50 and 59 hours for eight respondents, between 60 and 69 h for five, and over 70 h for two participants.

Organization-specific bureaucratic requirements were most frequently rated in the range of up to 10% of own working time (*n* = 6), whereas sector-specific bureaucratic requirements were more frequently in the range of 11–20% (*n* = 6).

The office was the most commonly reported workplace. Participants worked an average of 57% of their working hours in the office (SD: 26.65, range: 10–90). On average, 27.2% of working hours were spent working from home (SD: 24.03; range: 0–80%). Working outside the office was the least common location (range: 0–35%; M: 15.8; SD: 13.19).

The commuting time ranged from 10 min to 6.5 hours, whereby the majority of commuters traveled alone by car (*n* = 8) or by public transport (*n* = 6).

Participants reported an average of 25.47 years of experience in the sector (range: 9–25; SD: 9.99), 12.47 years in the organization (range: 2.5–33; SD: 9.15), and 10.07 years in their position (range: 2–26; SD: 6.81). Seven people indicated that they did not consider leaving the organization for another position within the next 10 years. Three respondents said they considered it, while four intended to retire within the next 10 years.

#### 3.1.2 Hierarchical levels

The interviews indicate that participants occupy different hierarchical levels. Eight participants hold intermediary management positions between top management and operational level (*n* = 8; I04, I05, I08, I09, I10, I11, I14, I15), while six have hold a top executive management position (*n* = 6; I01, I02, I06, I07, I12, I16). Two participants hold two roles (*n* = 2; I03, I13).

#### 3.1.3 Health satisfaction

Although all participants rated themselves as healthy (inclusion criteria), the leaders assessed their satisfaction with their own health or personal health behavior in highly variable ways. Half of the participants reported high satisfaction with their health behavior. Three participants expressed low satisfaction with their health, the other leaders reported moderate satisfaction. Notably, a link to their working environment was predominantly observed in cases where dissatisfaction was reported.

### 3.2 Qualitative data: leaders´ understanding, causes and consequences as well as coping strategies of bureaucracy

Sixteen qualitative, guideline-based expert interviews were conducted with leaders from the health, education, and social sectors, yielding 12 hours and 15 min of transcribed material. The majority of participants were female (*n* = 10). The age group of 50 to 59 years (*n* = 7) accounted for the largest proportion of the sample (see Table 1).

The key findings on bureaucracy can be categorized into health-, emotion-, cognitive, leadership, inter-personal-, process-related and societal aspects, and are presented below in detail. The results encompassed the presented understanding of bureaucracy, *causes, consequences* as well as *coping strategies* and related *resources* within these categories.

All participants expressed at least some negative views on bureaucracy. Terms such as “bureaucratic monster” (I08), “bureaucratic jungle” (I12) and “bureaucratic madness” (I06) were used. One participant noted that the term bureaucracy “per se has an incredibly negative connotation” (I06). However, negative views of bureaucracy were also partially contextualized. For instance, one leader argued that while the term might evoke aversion, caution would be necessary due to its potential positive effects (I10). Positive perceptions of bureaucracy were mainly linked to the necessity of certain bureaucratic requirements (see Table 2).

**Table 2:** Leaders´ understanding of bureaucracy.

Categories	Leaders´ understanding of bureaucracy
Inter-personal	Sphere of authority Involvement of multiple individuals
Process	Framework conditions Overregulation Detachment of practical realities Absurdity
Society	(Lack of) meaningfulness

Managers referred to various forms to bureaucracy during the interviews. The processes and regulations mentioned as examples can be categorized into four groups: (1) bureaucracy aimed at establishing framework conditions (e.g., administration of financial resources, promotion regulations, procurement regulation, personnel matters, data management/security/protection); (2) bureaucracy in the sense of documentation (e.g., medical documentation; among other purposes for self-protection, creating transparency, and routine data collection); (3) bureaucracy as a mechanism of quality assurance through control (e.g., financial and societal responsibility to ensure compliance with laws or with a desired state of affairs, as well as quality checks); and (4) Application through bureaucratic processes (e.g., project applications, reimbursement of travel expenses, requesting access, initiating processes).

Participants identified a range of *causes* for bureaucracy (see Table 3). In some statements, a distinction is made between internal (organizational-specific) and external (e.g., politics) causes. One participant emphasized that external requirements might be influential in a more negative way, as they impact company development and risk, whereas internal requirements could often be more manageable.

**Table 3:** External and internal causes of bureaucracy.

Categories	External causes	Internal causes
Health	Need for control and safety	Need for control and safety
Leadership		Regulation Personnel matters Establishment of framework conditions
Inter-personal	Mistrust Fear Self-protection	Mistrust Fear Self-protection
Process	Data management Data protection Coordination requirements	Data management Data protection Coordination requirements Internal administration
Society	Financing Social responsibility Politics and legislation	Financing Social responsibility

Most of the described *consequences* of bureaucracy were negative. Although numerous negative effects of bureaucracy were emphasized, some participants also discussed positive outcomes, which, in certain instances, were given considerable importance (see Table 4).

**Table 4:** Negative and positive consequences of bureaucracy.

Categories	Negative consequences	Positive consequences
Health	Stress (mental and physical)	Inner sense of safety
Emotion	Frustration & misunderstanding Dissatisfaction Demotivation (no fun, boredom) Powerlessness & uncertainty	Satisfaction after completing
Cognition	Distraction (Loss of attention, Background noise)	Review the own work (careful handling of data)
Leadership	Boundaries in collaboration with employee Opportunity costs (time) mediation/handling employee frustration	Appreciation through transparency
Process	Restrictions Delays or prevention of progress High effort Tasks completed at the expense of substantive work Reduced quality	Structure Functionality Quality assurance Transparency
Society	Delineation Inhibition of innovation Lack of target group orientation Discontinuation of activities	Stabilization of society and democracy Legitimization of actions (Control of) enforcements of established regulations

Several respondents emphasized the importance of *coping strategies and resources* (see Table 5). They described resources as “a total source of strength” (I04), “a place of retreat” (I12), “vents” (I13). There was an acknowledgment that the demands of bureaucratic regulations tend to increase when resources are constrained due to private pressures (I15).

**Table 5:** Coping strategies and resources.

Categories	Professional environment	Private environment
Health		Exercise Setting boundaries Nature Sleep Nutrition Preventive medical check-ups
Emotion	Enjoyment of substantive work	
Cognition	Accepting Value-based integration Resigned compliance Behavioral disengagement Resource-oriented mindfulness attitude Time management	
Leadership	Communication (transparency) Coordination Protect/shield Simplify or bypass Active managerial role Trust Controlling results	
Inter-personal	Flexibility and solution-oriented approach of individuals Internal and external support	Family Friendships
Process	Creative leeway Mechanisms of evasion and refusal Internal change initiatives Exploring grey areas Organizational structures	
Society	Meaningfulness	

#### 3.2.1 Health-related aspects

The health-related *causes* of bureaucracy are partly due to a need for control and safety arising for instance from concerns about liability. This cause goes along with positive consequence of inner sense of safety, which could be enhanced by bureaucracy by fostering transparency and predictability in processes. Bureaucratic rules might provide clarity, guidance, and support for self-structuring, helping to manage risks and acting as a form of self-protection, particularly in large organizations.

However, the health *impact* of bureaucracy was a major concern for many interviewees. For instance, one participant noted that bureaucracy often complicates processes unnecessarily, hindering progress, which could, in turn, have negative effects on health. As a result, she experienced “incredible harm from bureaucracy” due to the communication challenges between employees (I16).

Most leaders reported that bureaucracy contributed to their experience of stress. It was described as a psychological stress factor, with some participants feeling that they had pushed beyond their limits to meet bureaucratic requirements. Bureaucracy was accompanied by a sense of pressure (e.g., due to expectations and unforeseen requirements), constant strain, and a feeling of losing control, all of which heightened stress levels. For instance, one participant noted that her stress could always be traced back to bureaucracy, not to her substantive work (I13).

Some leaders noted that while bureaucracy was an additional stressor, it was not the primary *cause* of stress. Instead, it was perceived as a situational stressor that could be the “last straw” (I06), particularly during periods of heavy workload: “breaks the camel’s back” (I06; I12; German: “Fass zum Überlaufen”). One participant also mentioned that bureaucracy could become more stressful when combined with personal crisis (I15). However, one leader emphasized that people often misattribute their stress solely to bureaucracy, overlooking other contributing factors such as being overworked. In such cases, bureaucracy would serve as a convenient excuse (I06).

Other participants discussed the physical stress of bureaucracy, citing symptoms like insomnia and physical tension (e.g., in the jaw and shoulder areas (I12)) or health issues from prolonged sitting (I14).

Health-related *resources* are found in the private environment, including factors such as exercise, setting boundaries, exposure to nature, sleep, nutrition, preventive medical check-ups.

#### 3.2.2 Emotion-related aspects

Most of the participants expressed feelings of frustration, agitation or annoyance, ranging from mild irritation to “it drives me crazy” (I06) in the context of bureaucracy. One leader described how bureaucracy could wear down one’s nerves: “Well, on a day like this, when there is a lot of bureaucratic work to do, I have to say, you are a bit more annoyed. Your nerves are very frayed on days like this” (I09). Bureaucratic processes were characterized as making work more challenging and becoming a “thorn” (I02) for employees.

The frustration might have stemmed from processes taking longer than anticipated, hindering progress due to the complexity of detailed regulations. One leader highlighted the difficulty of initiating processes and the frustration with numerous steps. Additionally, some participants cited the lack of mutual understanding between administrative and operational staff as a significant contributing factor. Bureaucracy was especially frustrating when facing uncooperative “nitpickers” (I04). Others reported that frustration often transformed into demotivation or boredom when the work lacked sufficient challenge.

Some participants described feeling helpless or powerless as bureaucratic demands increased and became more complex. Despite their efforts, it might not have been possible to keep up with all requirements, thereby diminishing their sense of self-efficacy and contributing to stress. For some participants, bureaucracy also might have triggered uncertainty, often arising when confronted with unfamiliar or infrequent requirements.

Dissatisfaction related to bureaucracy was frequently mentioned. Participants discussed how documentation did not align with their original motivation for pursuing their professions:

*So documentation in health professions is about 30 percent of our work. And for those who maybe want to have a career because they like caring for patients or operating or something like that or are nursing staff. We could have been writers. But that's what we are – we didn't do that on purpose (laughs).* (I08)

However, one participant expressed a strong sense of satisfaction after completing a bureaucratic process. For this person, this feeling was closely associated with a readiness to “get started” (I09). She also reflected on the long-term pride she might derive from well-documented processes.

Emotional *resources* are reported to be helpful in the general professional environment, including factors such as enjoyment of substantive work.

#### 3.2.3 Cognitive aspects

Cognitive factors appear to play a crucial role in how leaders categorize bureaucratic requirements. One participant emphasized the cognitive toll bureaucracy might take, describing it as a “constant background noise in the head” (I03), caused by the continuous switching between substantive and bureaucratic tasks, which might lead to distraction and fragmentation. However, some leaders emphasized that bureaucratic requirements could compel individuals to review their work, fostering more careful handling of data (self-check).

Cognition played a decisive role particularly in successfully *coping* with bureaucratic requirements. Accepting bureaucratic requirements, which cannot be changed, emerged as a predominant coping strategy among almost all participants. A closer analysis of the statement reveals that three distinct forms of acceptance can be identified: (1) value-based integration, (2) resigned acceptance, and (3) behavioral disengagement.

The value-based integration of bureaucracy means that bureaucratic demands may allow individuals to remain aligned with their personal values, such as supporting their team or ensuring the responsible management of public funds, in terms of the positive consequences of bureaucracy. Correspondingly, another leader perceived bureaucracy as his tool that he could use to achieve other objectives, such as enabling people with disabilities to be included in the labor market (I14).

The meaningfulness of bureaucratic demands played a crucial role in (value-based) accepting them. One leader mentioned that it might be easier to “put up with inconveniences” (I02) when one “can come to terms with the meaning behind them” (I02). For another leader, the same logic applied—if he perceived a process as “making total sense” (I15), then he would accept it and “drink it like clear water” (I15).

However, even in situations where a sense of meaning was absent, the demands were still accepted “grudgingly” (I12). If a process could be simplified, one manager might reflect on it for a bit longer, but as a civil servant, he remained bound by instructions. Even when he found certain regulations nonsensical, he would still implement them, as long as they did not violate the constitutional principles.

Leaders with this resigned acceptance used terms such as “arranging” (I08), “making friends with” (I15), “coming to terms with” (I15), or accepting the “conditions that I have to abide by” (I01) to describe their approach. Statements like “I accept what I have to do” (I01) further highlight this perspective. Similarly, some leaders regarded bureaucracy as a necessary means to an end. For instance, one participant described it as a “necessary evil [to] do what I really, really enjoy doing” (I13) and another participant metaphorically compared it to getting hit by a ball in a soccer match: “If you play soccer, you can also get hit by a ball, that’s how it is” (I15).

In some cases, leaders accepted bureaucracy only after their change initiative had failed, adapting by “go[ing] along with the forms” (I04). As one leader admitted, she could not “change the world” (I05). This reflection on one’s sphere of influence played a crucial role in managing these constraints.

In this sense, behavioral disengagement seems to be a successful coping strategy for many interviewees. This can be seen, for instance, in refraining from continuously questioning. Respondents highlighted that bureaucracy “is not to be questioned” (I03), because continuously questioning bureaucracy – opposed to accepting it – could have detrimental effects on health and motivation. For some, refusing to accept bureaucracy would “only lead to permanent misfortune” (I04), resulting in exhaustion or even a “collapse” (I15) or a “crisis” (I14). One leader provided a metaphor to illustrate this point:

*And then there are people who just don't accept that the system is the way it is and that you can't change it from where you are, on a large scale, but certainly on a small scale, and then you keep having these, in my opinion, problematic battles. And then you lose a lot of energy, you don't see what your role is, you get identity problems, you get existential crises, […], then it can happen that one or the other ´break down´ in this bureaucracy, right?* (I15)

Another way of cognitive coping with the burdens of bureaucracy can be a resource-oriented, mindfulness attitude. One participant illustrated this by advising not to oppose a negative event “raw” (I02), but instead to channel that energy toward constructive movement, inspired by aikido, a Japanese martial art. He also described maintaining a relatively calm demeanor in stressful situations. Other participants employed similar strategies. One leader emphasized the importance of “find[ing] her center” (I04) and distancing herself from actions of higher management. Another interviewee described adopting a stoic attitude during heightened stress, where he “steps out” (I10) of the situation, reflects, and determines the next course of action.

Regarding the time management of bureaucratic tasks, prioritization was the dominant technique, especially during periods of high work demands or when multiple tasks required completion. Other time management techniques mentioned included the “Eat the Frog” (I09), the use of calendars, resource planning, self-management based on one’s own resilience, timing, time buffers, scheduling breaks, and avoiding appointments on Fridays.

Further cognitive resources are located in the general professional and private environment, including factors such as awareness of their privileged position, self-efficacy, (self-)reflection, mindfulness, personal traits, and guiding principles.

#### 3.2.4 Leadership-related aspects

Leadership-related *causes* of bureaucracy include aspects such as regulation, personnel matters, setting frameworks.

Some leaders observed that bureaucracy might limit their capacity to engage with employees, particularly in matters such as salary negotiations or providing support for work-related tasks. Some interviewee noted a restrictive influence of bureaucracy on their leadership capabilities.

The leaders also discussed the opportunity costs associated with the substantial time required to manage bureaucratic tasks, which often left them with insufficient time for leadership responsibilities. One participant stressed the importance of effective resource management, explaining that she had to carefully allocate her time. Her statement, “how much effort I spend on bureaucracy and how much time and what is left over for leadership” (I07), underlines the tendency for bureaucratic demands to overshadow leadership duties. Similarly, another leader noted that leadership is sometimes deprioritized to comply with bureaucratic requirements.

Another impact of bureaucracy on leadership could be the need for mediating between different parties. One interviewee described the “translation role” (I16) she assumed between academic staff and administration, such as resolving issues related to travel expenses. She found this dual responsibility “quite exhausting” (I16). Similarly, another participant referred to her position as a “sandwich position” (I04). One participant noted that, although she occasionally struggled to empathize with employee complaints, she felt compelled as a leader to enforce bureaucratic requirements and take the “hard […] line” (I09).

However, one leader highlighted that the organization’s internal Human Resources program could track employees´ tenure, using this data to express appreciation during work anniversaries, demonstrating a positive application of bureaucracy for employee recognition.

All participants discussed *strategies* for managing bureaucratic demands to support health and motivation in their leadership roles.

Almost all leaders emphasized the importance of communication, particularly transparency. One leader noted how employees might often be less involved in bureaucratic processes, such as auditing, leading to a lack of understanding. As one participant explained, since bureaucracy typically has only negative effects on employees (e.g., increased workload, distraction), it falls to the leaders’ role “to explain the entire process to them” (I09). Transparent communication could provide clarity, enabling employees to work more independently and gain a better understanding of the processes.

Most leaders regarded the coordination of bureaucracy as a key leadership responsibility. Some emphasized that they did not need to address all requirements personally, but rather delegated tasks or established frameworks for specialized departments. Joint processing with employees or the management team was also seen as an effective approach. Being able to delegate recurring bureaucratic tasks rather than handling them personally was viewed as motivating, cost-saving, and contributing to overall satisfaction.

In contrast, some leaders preferred to “shield” (I02) their employees from bureaucratic pressure or at least “not burden them with it” (I12), aiming to “keep bureaucratic processes as far away from employees as possible” (I06). One leader explained that reducing external pressure could ensure that employees did not lose their “desire for further development” (I02).

Considering shortcuts was emphasized as a key leadership responsibility to simplify or bypass certain bureaucratic steps, such as conducting random checks or handling travel expense reports without receipts. This might have been particularly relevant in the public sector, where employees often struggled to “interpret existing rules generously or omit control loops” (I16), especially given the tendency for risk-averse individuals to be attracted to such environments. One participant described adopting an active managerial role, stating that she “partly also decides” (I08) which bureaucratic processes to implement. She perceived herself “in the driver’s seat”, rather than a “victim” (I08) of bureaucracy.

Trust was identified as a key leadership-related strategy for reducing bureaucratic effort. Inter-personal trust would enable leaders to leverage available discretion and streamline processes. However, this approach might have been hindered by ingrained habits and a rigid administrative mindset (I16). One leader adopted a leap-of-faith approach, letting employees decide whether to follow certain processes. He identified advantages in reducing the bureaucratic burden and giving employees more freedom. He often forwarded results “in the confidence that they will be good” (I03), relying on higher-level feedback if needed.

Controlling results emerged as another strategy participants might employ to manage bureaucracy, driven by various motives: (1) ensuring liability and responsibility through quality assurance, such as verifying plausibility and relevance, (2) safeguarding financial resources, (3) refining and adjusting processes, and (4) delegating tasks according to the personal resources of the team members.

#### 3.2.5 Inter-personal aspects

In terms of inter-personal aspects, some participants viewed bureaucracy negatively. One participant described bureaucracy as a “sphere of authority” (I04, German: “Herrschaftsbereich”), defined by the financial power of those who provide funding. Similarly, another leader referred to “omnipotence fantasies of the ministries” (I10). One interviewee associated the term bureaucracy with “dictatorship” (I01) and described certain bureaucratic practices as “redundant harassment” (I01). Another participant added that he believed the processes and timeframes at state universities were deliberately used to hinder progresses.

The involvement of multiple individuals in a bureaucratic process elicited mixed reviews. One participant noted that participation of others was viewed positively. Conversely, one participant highlighted that the involvement of different hierarchical levels, as well as other ministries and municipal associations, often contributed to delays. Due to the political sensitivity of decisions within the ministry, a chain of superiors was typically required, necessitating extensive coordination and making it difficult to “break out” (I15) of the hierarchy.

Mistrust, fear and the need for self-protection were frequently mentioned as inter-personal causes of bureaucracy. One participant attributed the perceived over-regulation of finances to a lack of trust in responsible fund management, describing it as a matter of trust (I10). Furthermore, one interviewee noted that neighboring departments often create bureaucracy “to protect themselves […]” (I15). Thus, a lack of trust or fear of making mistakes might contribute to increased bureaucracy. One participant added that individuals in administrative roles often adopt a more risk-averse mentality, which complicates “interpreting existing rules generously or omitting control loops” (I16). This “belt and suspenders strategy” (I16) stems from a fear of making mistakes.

In contrast, the flexibility and solution-oriented approach of individuals involved in various process steps was identified as a key resource. One leader found it beneficial to engage with “the key people” (I04) and establish shared goals before collaboratively addressing “the nitty-gritty of the forms and processes together” (I04). She emphasizes that people design and implement the rules (I04). Similarly, one participant highlighted the significance of inter-personal interactions in effectively navigating bureaucratic requirements (I12).

Further inter-personal *resources* are located in the general professional and private environment, such as organizational-internal (e.g., team) and –external (e.g., coaching) support, family and friendships.

#### 3.2.6 Process-related aspects

Most leaders described bureaucracy as a framework within their work environment. Terms like “rigid, clearly structured system” (I04), “regulated procedure” (I02), and “guideline” (I11) were used to emphasize this structuring function. Even in conceptual or creative tasks, such as writing a grant application, the process was said to be “never detached from a bureaucratic structure” (I10).

A recurring theme among participants was the notion of overregulation. While each bureaucratic requirement might be justified individually, the cumulative burden of excessive bureaucracy was said to demanded substantial human and material resources. Descriptions such as “too many steps” (I03), “too detailed overall” (I06) and “mammoth mess” (I08) were used. The depth of the regulations and the efforts applied were sometimes perceived as disproportionate to the resulting benefit.

One participant noted that in Germany, errors might lead to increasingly detailed regulations (I06). Another leader also described it as a “constant struggle not to let the bureaucracy over-shape the goal” (I04). According to another leader, one must “be very careful that it [bureaucracy] does not become a self-fulfilling wheel” (I01).

Several participants felt that bureaucracy might be detached from practical realities. For instance, one participant observed that bureaucratic processes were often said to be “not thought all the way through” (I07), even when well-intentioned. Another interviewee noted that certain draft laws could be “overtaken by reality” (I15), highlighting that the legislation often fails to reflect practical needs. According to another leader, bureaucrats might not always consider “what practical effort this would entail and how far removed from reality it is” (I08). She also emphasized that many forms were often said to be inaccurate or not individualized. One participant called for a stronger coordination process between the legal and practical authorities, as those responsible in the Ministry of Education were often perceived as disconnected from the “front line” (I10).

Some participants described certain bureaucratic regulations as absurd in both their extent and form. For instance, one participant highlighted the process of obtaining a doctor’s note as “completely absurd” (I02). He pointed out the absolutism of requiring a doctor’s note without any gradations, as well as the necessity for employees to certify illness through a doctor. Similarly, another interviewee referred to these as “absurdities” (I12), noting financing requirements of public tenders might be particularly problematic.

Process-related *causes* of bureaucracy include factors such as data management, security, and protection, particularly with regard to health data, as well as coordination needs. Internal factors also play a role, including internal administration.

The restrictive nature of bureaucracy was a key focus of the process-related *consequences*. Bureaucracy was described as “continually slowing people down” (I09). Another participant, for instance, explained how bureaucratic procedures restricted decision-making in business contexts. Additionally, self-imposed limits, such as promotion regulations, were also seen as obstacles, even though some degrees of freedom were incorporated in their design (I06).

The delay or prevention of progress was viewed as a key aspect of bureaucracy’s restrictive impact. For instance, one leader described how bureaucratic requirements in the healthcare system complicated processes. Another participant used the metaphor of “two speeds” (I03) – the bureaucratic pace versus the pace of the modern working world. He explained:

*And whenever I have to rely on specifications and predetermined process paths, I have to take the back road, so to speak, and have to stop at every milk can, because it is planned that way, that you have an additional coordination meeting somewhere here in the building.* (I03)

He and others believed that processes could be accelerated while remaining “quite safe” (I15). Delays often arose due to the large number of actors involved.

The effort involved in bureaucratic processes was another source of stress. Overall, bureaucracy was perceived as time-consuming and resource-intensive. Participants highlighted the negative impact of having to repeat processes (e.g., follow-up regulation, re-examinations, incorrect forms, changes of framework conditions or the specification of earmarked funding projects). Bureaucracy often required multiple work loops, with some leaders needing to “keep a close eye on the process” (I10), while one described it as a “never-ending tail” (I09; German: “Rattenschwanz”), having to track whether other units responded or handled the process correctly.

About half of the respondents discussed how bureaucracy negatively impacted their substantive work. Some felt that bureaucratic requirements diverted time away from essential tasks, such as providing support to those in need. As one participant stated, after dedicating time to bureaucracy, focus would shift back to the “actual processes” (I07). Others viewed bureaucracy as an additional burden in an already demanding job, contributing to emotional effects like irritability.

Highly detailed regulations could result in reduced quality or in bureaucracy that “misses the point” (I11). For instance, procurement regulations might prevent the best job applicant from being selected. Bureaucratic processes could also stifle “your own creativity and flexibility” (I04). One participant mentioned a “data graveyard” (I08) created by the extensive documentation requirements, questioning whether this data was ever actually utilized. Conversely, several participants emphasized that certain bureaucratic processes could ensure functionality and maintain quality, particularly during crises or for purposes such as insurance benefits. Routine data collection, quality checks, and process adjustments might contribute to improving quality and identifying “black sheep” (I05). For instance, ensuring evidence-based medicine or adhering to good scientific practices often requires data collection, which might only be feasible through bureaucratic means.

Further positive, process-related consequences are the structuring function and the resulting transparency. Bureaucracy can provide “support, structure, and clarity” (I01), which is linked to an inner sense of safety. These structures might assist employees, even if they experience bureaucracy as frustrating. Transparency, often regarded as a positive aspect of bureaucracy, was highlighted in the context of the responsible use of public funds and personal traceability. Proper documentation could strengthen traceability and accountability, ensuring that processes and decisions are well-documented and subject to review.

Process-relates *resources* - like creative leeway, mechanisms of evasion and refusal, internal change initiatives, and exploring grey areas/pragmatism were also accorded high priority.

Creative leeway and the organizational framework were crucial for the leaders. Almost all participants highlighted self-efficacy, in the sense of the belief in one’s own ability and creative leeway to manage tasks, as a key resource, often linked to co-decision making, or decision-making freedom: “What keeps me in this field of work is the self-efficacy and the opportunity to shape and organize and also to influence processes well. (I07).

Despite bureaucratic constraints, having creative leeway, decision-making power, the ability to manage processes and experiencing self-efficacy was viewed as essential. As one participant stated:

*I think that, first and foremost, as far as the professional component is concerned, it is the experience of self-efficacy and autonomy. I think that it is absolutely crucial. Well, we are talking about bureaucratic structures, but they do not prevent self-efficacy. So, in my opinion, it is absolutely important to be able to proactively shape the work you are doing, within a certain framework of course.* (I10)

Some participants described mechanisms of evasion and refusal when they deemed bureaucratic regulation to be unnecessary. For instance, one participant referred to a Federal Labor Court ruling on working time recording, which he might consider superfluous as a managing director. He complies by “rais[ing] his hand once a month to show that I have looked at [the] working hours” (I06), noting that such processes add “no real value” (I06) since behavior does not change. He mentioned refusing to adhere to certain documentation requirements. Instead, he assumed personal liability as a managing director.

Refusing bureaucratic processes could also prompt internal change initiatives. One respondent described refusing to print, sign, and rescan a service ID card. She would find it more efficient if she were authorized on the basis of her e-mail request (I07).

Exploring grey areas could have been another strategy for managing bureaucratic requirements. Participants identified shortcuts, described as a situation-appropriate “path B” (I03). One participant emphasized that understanding the system and drawing on professional experience could help navigate these shortcuts effectively. Similarly, one participant noted the importance of not taking bureaucratic requirements “too seriously” (I10) to minimize effort and maintain efficiency (pragmatism).

The organizational structure was also a resource from the general professional environment for some participants.

#### 3.2.7 Societal aspects

For one participant, the term bureaucracy was closely associated with a lack of meaningfulness. Another leader emphasized that every bureaucratic requirement should ideally lead to a positive outcome, such as improving speed, quality, or customer and employee satisfaction.

When formal requirements were seen as meaningless or counterproductive, bureaucracy was perceived negatively. Terms such as “nonsense” (I08), “this stupid, superfluous thing” (I08), “the most senseless things” (I12), and “utter rubbish” (I15) were used to describe regulations.

In the context of the societal aspects, the (lack of) meaningfulness played a major role, both in the leader’s *understanding* of bureaucracy, but meaningfulness also represents a *resource* in itself. Leaders reported that bureaucracy is more manageable when they perceived the task as meaningful. One leader explained that, even when she does not fully agree with the regulations, understanding the underlying rationale made it easier to handle the task (I08). For instance, ensuring transparency and legitimizing the use of public funds as well as adhering to data protection regulations – particularly within the healthcare system and in the context of awarding educational qualifications – were considered valuable. Another interviewee shared that she can better handle rejections when they were not seen simply as a “demonstration of power” (I04; German: “aus so einer puren Herrschaftsgeste raus”). Similarly, one participant emphasized the importance of communicating the meaning and purpose of regulations to her team (I11).

Societal *causes* of bureaucracy include factors such as financing and societal responsibility. External factors also contribute, including politics and legal frameworks as well as sector-specific features and legal structures.

Bureaucracy might often reinforce the delineation of separate work areas, potentially hindering the achievement of “desirable target states” (I04). For instance, one participant noted that bureaucracy might encourage “working in gardens” (I04). The imagery of gardens reflects the segmented nature of organizations, a structure seen as impractical compared to the realities of work. A holistic approach, such as a unified data protection agreement, was viewed as more effective. Another participant highlighted a “silo view” (I02) within the healthcare system, where different actors like doctors, physiotherapists, and pharmacies were trained to focus solely on their own areas, further reinforcing bureaucratic fragmentation. These requirements promote the implementation of “the current structures in the healthcare system, in the respective silo view” (I02). Additionally, one leader discussed the strict demarcation between hospital and outpatient care in Germany, particularly in discharge management (I08).

The challenges at the interfaces of different working areas in the educational sector, such as the transition to the next educational institute or exchange between educational institutes in different federal states, caused by bureaucratic requirements, were also discussed. In particular, data management issues, such as the inability to effectively process collected data (e.g., insufficient knowledge transfer between kindergartens and schools or across federal states), were identified as “one of the biggest bureaucratic hurdles” (I12). Additionally, the bureaucratic separation between care and educational institutions further impedes achievements of educational success. Different sponsors might contribute to “bureaucratic incomparabilities” (I12), which are exacerbated by inconsistencies between state and federal programs.

Bureaucracy could also stifle innovation and impede agility, as one participant described for work within ministries (I15). A lack of openness to new ideas could often slow progress. As society evolves rapidly, government processes frequently might struggle to keep pace to societal innovation, as described with the following metaphor: “as a government, you have to be careful not to ride the horse and the others are already flying” (I15). Resistance to change and “[hiding] behind a lot of bureaucracy” (I15) were identified as critical barriers.

Some participants argued that bureaucratic regulations might frequently be misaligned with the needs of the target groups. One participant predicted that data protection could become the leading cause of death in the future, emphasizing that patients might likely consent to the use of their data if it provided tangible benefits (I06). Another participant highlighted inefficiencies in hospital discharge management.

Bureaucracy could lead to the discontinuation of activities, particularly when leaders become frustrated or overwhelmed by administrative burdens. One participant noted that bureaucracy in Germany might act as a deterrent to entrepreneurship.

These negative, societal *consequences* are closely linked to the positive effects at the societal level.

Some participants argued that bureaucratic processes could create a societal foundation that ensures stability. The role of bureaucracy in maintaining democracy was also emphasized, with participants suggesting that a world devoid of bureaucracy might be unjust and chaotic.

Several interviewees, particularly those working with public funds, felt a responsibility to justify their actions through bureaucratic requirements. As one leader stated:

*It is our duty to handle public funds properly and transparently. So if certain guidelines for the expenditure of funds, if certain processes for ensuring transparency are apparently necessary, then I would always tend to say that's just the way it is. And we are obliged to legitimize our actions and always put ourselves in the service of our public mandate […].* (I10)

Another added that “efficiency and economy” (I16) must always be taken into account when using tax money.

It was further mentioned that bureaucratic control could ensure the enforcement of established regulations, such as European Union standards on child labor and occupational health and safety. This auditing, along with internal adherence to external rules, was viewed positively.

### 3.3 Second quantitative survey

Fourteen participants (87.5% of the sample) took part in the second survey. The majority of participants were female (n = 8), with the age group of 50–59 years (n = 6) being most strongly represented in the sample (see Table 1). All respondents indicated that they were in a similar professional and personal situation as at the time of the interview.

The statement “I accept bureaucratic requirements, even if I do not consider them meaningful” showed a slight tendency toward agreement (=accepting) (M: 3.57, SD: 1.05). Continuously questioning of bureaucratic requirements was, according to respondents, generally perceived as having a negative impact on motivation (M: 3.79; SD: 1.26) rather than health (M: 3.07; SD: 1.44). A potential connection between bureaucratic requirements and a cynical attitude toward the job was generally not agreed upon (M: 2.14; SD: 0.91). Participants mostly rated the statement that insecure employees benefit from bureaucratic regulations as *partially true* (M: 3.14; SD: 0.74). In contrast, there was strong agreement with the statement that a better error culture would reduce uncertainty (M: 4.43; SD: 0.82).

**Figure 3:**
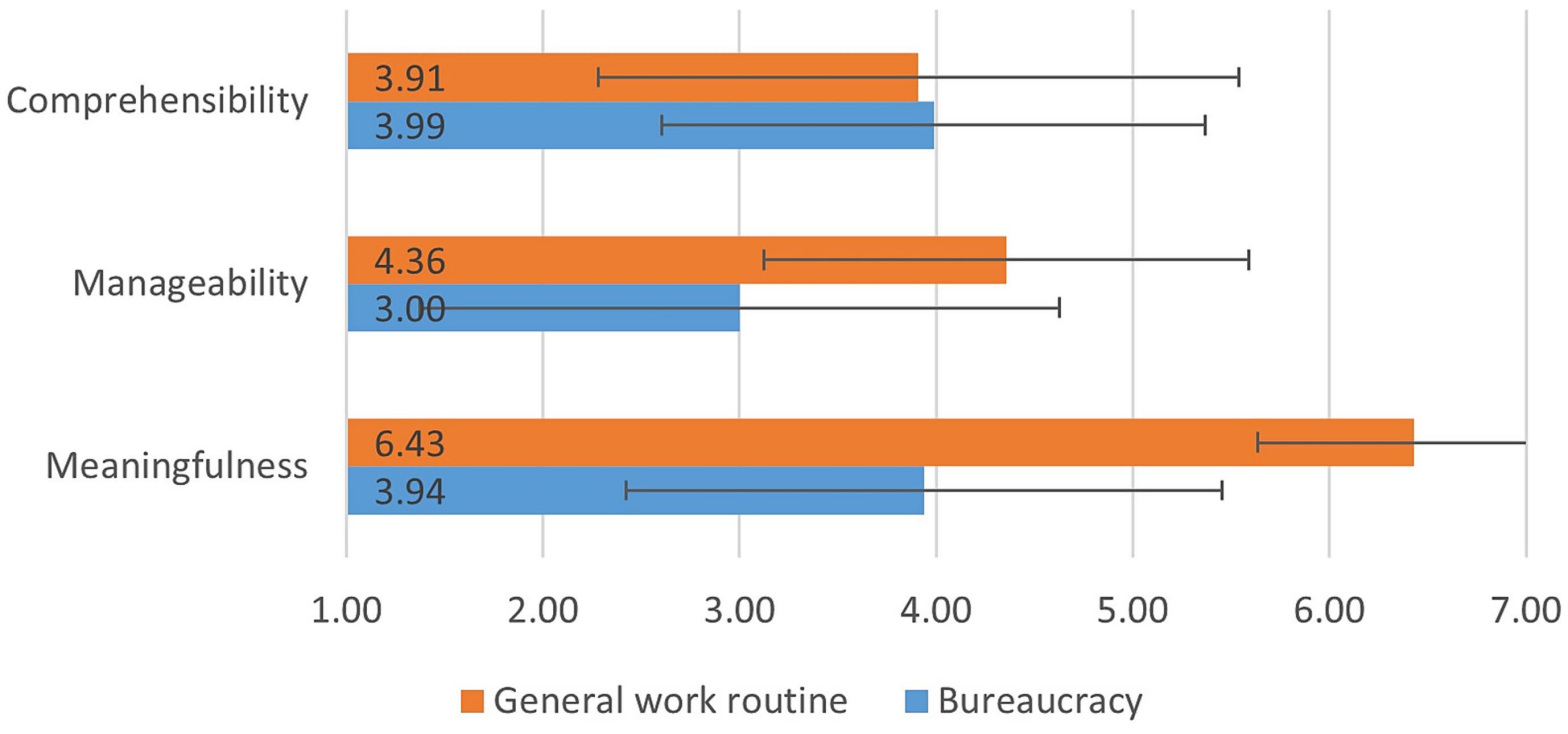
Bar chart of work-related sense of coherence, general work situation and bureaucracy, *n* = 14.

Overall, participants exhibited a strong work-related *SOC* (see Figure 3). The subscale for *comprehensibility* in general work situations had the lowest mean of 3.91 (SD: 1.63) and was comparable to the *comprehensibility* of bureaucratic requirements (M: 3.99; SD: 1.38). The *manageability* of the general work situation received an average rating of 4.36 (SD: 1.23), while that of bureaucracy was much lower on average, at 3.00 (SD: 1.63). The most significant discrepancy in the evaluation of *meaningfulness* was found between the general work routine (M: 6.43; SD: 0.79) and required bureaucracy (M: 3.94; SD: 1.52), with the scale reaching the highest value for general work demands.

The sense of belonging to the team (M: 4.48; SD: 0.6), sector (M: 4.38; SD: 0.86), and organization (M: 4.37; SD: 0.73) was comparably high.

## 4 Discussion

In this study, leaders from the health, social, and educational sectors in Germany presented diverse perspectives on bureaucracy. Bureaucracy was perceived neutral as a framework, negatively as a sphere of authority and positively as a source of an inner sense of safety. Particularly, the experience of a lack of meaningfulness regarding bureaucratic regulations were described as obstructive.

*Causes* and *consequences* of bureaucracy as well as *coping strategies and resources* of leaders in this working environment were identified. Participants frequently distinguished between health-, leadership-, inter-personal-, process-, and societal-related causes of bureaucracy. The reported effects of bureaucracy were predominantly negative. For instance, health-related consequences included psychological stress and physical tension; process-related effects involved structural inefficiencies, reduced quality, and compromised functionality. To cope with these challenges, the leaders employed a range of strategies and utilized various resources. For instance, change initiatives were emphasized as necessary coping mechanism along with various forms of acceptance (value-based integration, resigned compliance, behavioral disengagement) of specifications.

The modern working world and bureaucratic processes were frequently described as operating at two different paces, highlighting the need for better alignment between them. Several participants advocated for a culture that embraces learning from failure rather than relying heavily on rigid regulations. They emphasized that even the most detailed regulations cannot fully account for the complexity of real-life system and cannot entirely prevent errors.

### 4.1 Context within existing literature

The participants´ perception of a growing bureaucratic burden over time is supported by federal data. From 2014 to 2024, the number of federal laws in Germany increased from 1,671 to 1,792, with individual regulations increasing by almost 18% (German Federal Parliament, 2024).

Although the axial model of Grounded Theory postulates a linear relationship between causes and consequences, the data suggest that a certain circularity of processes can be assumed. Organizational theories show that actions, interpretations, and structures mutually influence one another (Weick, 1995).

Perceptions of over-bureaucratization were common among participants in the health, education, and social sectors. Nonetheless, many acknowledged the intended benefits of these regulations. According to van Loon et al. (2016), rules can be classified based on two criteria: (1) the extent to which they fail to achieve their intended goals, and (2) the burden of compliance (e.g., time, energy). When both criteria apply, administrative science speaks of *red tape* (“ineffective but burdensome rule” (Kalucza and Hattke, 2020)). Kalucza and Hattke’s meta-analysis (2020) demonstrated that *red tape* is associated with reduced satisfaction and lower commitment. In van Loon et al.´s study (2016), approximately 20% of rules related to core tasks were classified as *red tape* by public sector employees, whereas this figure was twice as high for human resources and financial tasks.

In contrast, about 30% of the regulations related to core tasks were classified as *high-quality rules* — neither lacking functionality nor creating unnecessary hurdles in implementation. However, this percentage dropped significantly for human resources (18.9%) and financial tasks (16.7%) (van Loon et al., 2016). The authors suggested that employees find greater meaning in the bureaucratic processes related to their core tasks than in those associated with human resources and financial tasks. Therefore, they stressed the importance of communicating the purpose behind regulations within organizations (van Loon et al., 2016).

A sense of meaningfulness emerged as a critical resource for maintaining motivation. Previous research has shown that promoting meaningful work is associated with greater engagement (Geldenhuys et al., 2014; Lee et al., 2017), which in turn enhances organizational commitment (Geldenhuys et al., 2014). Meaningfulness also improves goal achievement, reduces turnover intentions (Kipfelsberger et al., 2022), and enhances life satisfaction while reducing emotional exhaustion (Kim and Beehr, 2018). Positive effects on the perceived meaningfulness of one’s work can be fostered by leadership approaches such as empowering (Lee et al., 2017), resonant (Wallkamm, 2024) or visionary styles (Kipfelsberger et al., 2022). Most respondents in the present study emphasized the importance of transparency in their leadership roles. In particular, transformative and employee-focused leadership, along with high-quality interactions between leaders and team members, were considered beneficial for mental health. This is supported by occupational science (Pundt et al., 2018).

It was notable that bureaucracy and substantive work were often seen as coexisting. Some participants framed their entire work within bureaucratic processes, while most viewed bureaucracy as an additional task. The leaders expressed a high level of affective commitment to their substantive work, which, according to the literature, is associated with a more positive mood, reduced cynicism, and lower turnover intentions (Gillet et al., 2015). This commitment also correlated with higher job satisfaction, increased performance (Kustiawan et al., 2022), greater work engagement, and reduced feelings of personal failure (Kuok, 2022). The negative perception of bureaucracy, compared to substantive work, may be explained by the lack of affective commitment to bureaucratic tasks.

The distinction between substantive work and bureaucratic tasks may also partly explain the differing effects. Karasek’s (1998) *Job Demand-Control Model* categorizes work into four types based on two criteria: (1) the amount and nature of demands and (2) the controllability of the work task. Activities with high demands and high controllability (*active job*) are classified as conducive to learning and motivation, whereas those with high demands but low controllability (*high strain job*) are associated with risks to mental and physical health. Based on the interviews, substantive work appears to be perceived as an *active job*, while bureaucracy could be classified as a *high strain job* or a *passive job* (low demands, low controllability). Karasek surmised that the constant repetition in *passive jobs* could pose a lack of intellectual challenge, which could lead to a gradual reduction in problem-solving skills, boredom and dissatisfaction (Kain and Jex, 2010).

In occupational science, the negative effects of demands on health and the positive effects of motivational resources have been well researched and are represented in the *Job Demands-Resources Model* (Bakker and Demerouti, 2007; Demerouti et al., 2001; Demerouti and Bakker, 2011). In this model, bureaucracy – as described by the participants – could be considered a demand, particularly regarding health, where its effects were largely negative, especially when it intensified existing stress. Creative leeway in substantive work served as a motivational resource, while it was largely absent in bureaucratic tasks. A decisive factor in assessing these demands is the degree of self-determination and the sense of self-efficacy (Althammer and Michel, 2012). Studies clearly show that a lack of creative leeway negatively affects job satisfaction (Burgard and Lin, 2013).

The participant’s perception that disproportionate effort of low-meaning tasks intensifies negative effects aligns with existing research, which indicates a higher risk of burnout when a *SOC* is not strongly pronounced (Antonovsky, 1987, 1993; Eriksson and Lindström, 2006). The findings of González-Siles et al. (2022) reveal that *SOC* is negatively correlated with stress, depression and burnout. In contrast, *SOC* is positively correlated with job satisfaction, well-being and quality of life. The authors conclude that *SOC* may serve both as a mediator and predictor of these health outcomes (González-Siles et al., 2022). A strong *SOC* — associated with less emotional exhaustion, lower depersonalization, and higher personal performance and work commitment — supports stress coping, as demonstrated in a study with nurses in South Africa (van der Colff and Rothmann, 2009). *SOC* enabled the nurses to adopt more active, problem-solving strategies, which predicted lower burnout levels and high commitment (van der Colff and Rothmann, 2009). Leaders in our study described the dimensions *comprehensibility* and *manageability*. However, the dimension *meaningfulness* appeared less prominent in relation to bureaucracy. Moreover, the results of the second survey emphases the meaningfulness of general work activities was rated very high, whereas the meaning of bureaucratic tasks was rated significantly lower.

Although the *SOC* is a valuable model for understanding health and stress coping, there are some challenges and criticisms in practice. Current research suggests that the distinction between these three dimensions is not undisputed. A lack of isolation between the dimensions may be associated with measurements issues and should be critically examined further (Eriksson and Contu, 2022).

In dealing with bureaucracy, leaders employed various coping mechanisms, which can be categorized into the three engagement levels of the seven-stage Behavior Change Resource Model by Michaelsen and Esch (2021, 2022, 2023). All forms of accepting and refusal fall into *non-engagement*, while *motivational engagement* involves reflecting on existing processes or considering alternatives. The actual change initiative corresponds to the *executive engagement* in the model. If the change initiative fails, accepting (resigned compliance) bureaucracy serves as a functional fallback.

Accepting bureaucratic requirements play a crucial role in maintaining health and motivation in the health, education, and social sectors. Research supports that accepting such demands can reduce the negative effects of stressors, contributing to better mental health outcomes, such as lower levels of depression and anxiety. This coping strategy is particularly effective in work environments with low autonomy (Britt et al., 2016). Since bureaucracy is often associated with tasks that entail low autonomy, this appears relevant in this context. Moreover, a randomized controlled trial among healthcare workers found that increasing mindfulness contributed to significant reductions in psychological stress and burnout (Prudenzi et al., 2022). This can be an indicator, that accepting of unchangeable conditions and regulations (as a part of mindfulness) can help mitigate negative effects. The results of the second quantitative survey confirmed the thesis from the interviews that continuously questioning bureaucratic requirements was perceived as potentially having a negative impact on motivation and (to a lesser extent) health. Although accepting of bureaucratic requirements was described as a key coping strategy, which could indicate a distancing from the job, leaders stated that they had neither become more cynical nor doubted the meaningfulness of their work due to bureaucratic requirements.

Throughout the interviews, leaders mentioned various resources from their professional and private lives, including spiritual and work-cultural aspects. The relevance of these factors was also mentioned in Listopad et al. (2021b) as protective factors for burnout. For instance, the authors highlight that assessing the meaningfulness of tasks (such as bureaucracy) serves as a crucial resource in accepting the additional effort required (Listopad et al., 2021b). The authors proposed an extension of the bio-psycho-socio model (Engel, 1977) to enable a holistic understanding of burnout. Within the scale of perceived meaningfulness proved in the investigation of Listopad et al. (2021a) the positive meaning as largest beta coefficient for both burnout and work engagement.

### 4.2 Limitations

The study has methodological limitations that constrain the generalizability of its findings. The primary analysis is based on qualitative data collected from a small sample (n = 16), which is not representative of the broader population. However, the aim was not to achieve statistical representativeness but rather a strong information power (Malterud et al., 2016). The findings provide a solid foundation for further quantitative research, as we have reached a strong information power.

The inclusion and exclusion criteria relied on self-assessment, introducing potential subjective and social desirability bias, as maintaining an appearance of health and motivation is socially desirable in challenging working environment. Among other factors, the high heterogeneity of the sample can be attributed to the self-assessment of the leaders. It should not be overlooked that this heterogeneity in the self-assessment of health status limits the explanatory power regarding which coping mechanisms support the maintenance of health. More detailed inquiries into health have indicated that these perceptions are somewhat restricted. Based on the seven-stage behavior change model by Michaelsen and Esch (2021, 2023) outlined above, the leaders could be classified to be at varying stages of change. While all demonstrating awareness of the relevance of health-promoting behaviors (stage 2), only a few had reached the maintenance stage (stage 7).

Additionally, women were overrepresented (62.5%), despite men typically holding the majority of leadership positions. This gender imbalance may have introduced biases.

Participants came from various hierarchical levels, which may have influenced their involvement in bureaucratic processes and their relationships with employees. This heterogeneity could affect the comparability and consistency of the findings, as participants’ experiences and perceptions likely varied depending on their rank.

Furthermore, the present study conceptualizes bureaucracy primarily as an organizational stressor. However, it should be acknowledged that a critical perspective consider bureaucracy as an expression of power and control structures (e.g., Courpasson, 2000; Graeber, 2015), as also reflected in the participants´ accounts (see inter-personal aspects). As the research question primarily focuses on the salutogenic coping with bureaucracy, no more in-depth theoretical positioning was undertaken in this regard.

The recruitment process also presents potential limitations. Most participants were recruited through personal contacts and recommendations (convenience sample, n = 6; gatekeeper, n = 4), while additional participants (*n* = 6) were identified through targeted research. Since generalizability was not the objective, this pragmatic approach was considered appropriate.

Data collection was conducted via video conferences. Although this method is common for the target group, it may have limited the development of rapport between interviewer and interviewee, potentially influencing the responses. However, a positive aspect is that participants may have felt more comfortable being interviewed in their familiar environment.

The interpretation of the data should be considered in the context of the interview guidelines. While the subject areas were pre-determined, the questions were designed as open-ended as possible. The interview guide was developed with input from a qualitative analysis group, ensuring it addressed the relevant topics. Although the guide provided some level of standardization, it remained flexible to account for the time constraints and individual preferences of the participants. The iterative nature of *grounded theory* influenced the process, with earlier interviews informing subsequent ones.

### 4.3 Future research

The field of research is still emerging, but appears to be highly relevant to workplace health promotion. Due to the limited theoretical foundations currently available, further research is essential. Quantitative studies investigating the effects of bureaucratic structures and health promotion interventions in bureaucratic work environments would contribute to more comprehensive and generalizable findings. Including physiological measures in future, studies would also provide a more in-depth-analysis of how bureaucratic processes impact health. Since the small sample size did not allow for sector-specific-differentiation, future research should explore these differences.

Moreover, it should be investigated whether the positive effects of bureaucracy on leaders, in terms of inner sense of safety and process clarity, could be achieved through other means, such as a health promotion intervention or a modern organizational learning and error culture. If this were the case, reducing bureaucracy in favor of lower workload or a stronger focus of substantive work could be considered.

While the health promoting effect of mindfulness, among others acceptance without judgment (Kabat-Zinn, 1998) has been confirmed in numerous studies (e.g., Jayewardene et al., 2017; Khoury et al., 2015; Michaelsen et al., 2023), the extent to which acceptance can lead to positive health outcomes, even in cases of negative evaluation, requires further investigation. Additionally, acceptance with negative evaluation as a potential predictor of cynicism — a known risk factor for burnout — should also be examined. Interviewees generally assumed that this attitude toward bureaucracy was less harmful to their health compared to continuously questioning it.

The hypothesis that leaders who expressed satisfaction with their health (behavior) did not perceive a connection to their employment, whereas those who were dissatisfied attributed their dissatisfaction to the demands of their work life, requires further testing through a quantitative study with a larger sample size.

## 5 Conclusion

Leaders from the German health, education and social services face work-related demands that frequently lead to stress. Bureaucratic requirements are central in these industries and are often associated with negative impacts on health and motivation. These effects are particularly pronounced when bureaucracy is perceived as disconnected from core substantive work, when the additional time and effort required are deemed unjustified, or when resources are insufficient to manage the workload effectively.

To cope with these demands, leaders rely on a range of resources — whether professionally or privately. Utilizing leadership influence to improve bureaucratic regulations and accepting unchangeable bureaucratic demands were seen as crucial for maintaining motivation and health. In contrast, behavioral disengagement — contrary to accepting in the sense of value-based integration — was perceived as potentially stressful and psychologically limiting.

In conclusion, bureaucracy provides necessary structure and clarity in complex work environments. However, it is often perceived as restrictive and time-consuming. The inner sense of safety derived from process clarity can be regarded as a valuable health resource. Similar positive effects could be achieved through a trust-based culture that promotes learning and tolerance for mistakes. The benefits of such a culture are likely to complement the positive aspects of bureaucracy, potentially mitigating its negative impact. A multidimensional strategy of bio-psycho-socio-spirito-cultural factors — such as redesigning bureaucratic processes and fostering a culture of learning — should be further explored to enhance employee health and improve work outcomes.

## Data Availability

The raw data supporting the conclusions of this article will be made available by the authors, without undue reservation.
