# Supplementary material for: Between acceptance and change – a qualitative study about how leaders deal with bureaucracy
Source: Front Psychol. 2026 May 12;17:1739144. doi: 10.3389/fpsyg.2026.1739144 (PMC13201172; doi:10.3389/fpsyg.2026.1739144)
Supplement: Supplementary file 1 [file Table_1.docx]

Supplementary Material

# Supplementary Material 1: Final Category System

| **Category [Definition]** | **Sub-category** | **Definition (Sub-category)** | **Example quote(s)** |
| --- | --- | --- | --- |
| **Leaders´ Unterstanding of Bureaucracy**  [This category includes interview excerpts addressing leader´s understanding of bureaucracy.] | Inter-personal | Bureaucracy is understood in relation to inter-personal aspects, including the understanding of bureaucracy as a sphere of authority | - “At the same time, bureaucracy is a sphere of dominion because, to put it bluntly, they hold the money” (I04) - “[…] I sometimes like to use terms such as ´delusions of omnipotence´ of the ministries, which really bother me […]” (I10) |
|  | Process | Bureaucracy is understood in relation to processes, including framework conditions, means to an end, overregulation, detachment of practical realities, absurdity, and the involvement of multiple individuals. | - “The entire system is bureaucratically structured.” (I10) - “I perceive it as a necessary evil to do something I really, really like to do” (I13) - “The regulations, […], I consider them not goal-oriented but overly regulatory” (I01) |
|  | Society | Bureaucracy is understood in relation to its societal aspects, including its (lack of) meaningfulness. | - “Every single one of these bureaucratic requirements is legitimized” (I04) - “The sense of being unnecessary mainly arises from the thought that we could make this much simpler or, in some cases, even omit it” (I03) |
| **Causes of Bureaucracy**  [This category includes interview excerpts addressing the causes of bureaucracy, both internal (such as organizational) and external (such as political).] | Health | Causes of bureaucracy related to health aspects, such as the need for control and safety. | - “[…] I can of course understand the need for control and safety on the part of an association´s leadership, which says: ´We are holding the organization together. We […] are personally liable´. These risks need to be managed.” (I04) |
|  | Leadership | Causes of bureaucracy related to leadership-related aspects, including regulation, personnel matters, and the establishment of framework conditions. | - “Sometimes, however, there is also a bureaucratic area, for example, a budget allocation request or year-end financial statements. These affect all departments. I direct them to the […] administration department […]” (I07) - “These are partly standardized processes, […]. It can be something very small, like a vacation request, all the way to hiring.” (I10) |
|  | Inter-personal | Causes of bureaucracy related to inter-personal aspects, such as mistrust, fear, and self-protection. | - “So, the Social Code book five does include provisions for freedom, but they are interpreted so narrowly, […] and so fearfully interpreted […]” (I02) - “So, often you also have the feeling that there´s some kind of distrust on every side, and you just have to document everything accordingly” (I08) |
|  | Process | Causes of bureaucracy related to processes, including data management, data protection, internal administration, and coordination requirements. | - “For example, to ensure data protection or data security” (I08) - “[…] We also have administrative bureaucracy concerning the organization of staff, the organization of locations, and the duty rosters” (I01) |
|  | Society | Causes of bureaucracy related to societal aspects, such as politics and legislation, funding, and social responsibility. | - “So generally, these are laws or law-like regulations that we have to comply with” (I16) - “So, when certain requirements for the expenditure of funds, when certain processes for ensuring transparency seem to be necessary […]” (I10) |
| **Effects of Bureaucracy**  [This category includes interview excerpts addressing both positive and negative effects of bureaucracy.] | Health | Health-related effects of bureaucracy, including stress, physical health, and psychological safety. | - “But I believe that [bureaucracy] can be the last straw that breaks the camel´s back” (I06) - “Yes, and otherwise, bureaucracy also provides security, that has to be said” (I15) |
|  | Emotion | Emotional effects of bureaucracy, including frustration & misunderstanding, dissatisfaction, demotivation (no fun, boredom), powerlessness & uncertainty. | - “So, on a day when a lot of bureaucratic tasks are on the agenda, I have to say, you’re definitely a bit more irritated. The patience is very thin on such days” (I09) |
|  | Cognition | Cognitive effects of bureaucracy, including loss of attention, distraction, and background noise. | - “So, many things that ultimately create a constant background noise in the mind and cause constant switching, and especially many distractions” (I03) |
|  | Leadership | Leadership-related effects of bureaucracy, including boundaries in collaboration, opportunity costs (time), mediation, handling employee frustration, and appreciation through transparency. | - “[...] This translation function that one has as a leader is quite exhausting, often. Because you see how, practically, on both sides […] this really wears people down on both sides, right? And you have to try to keep both sides engaged and motivated despite this.” (I16) |
|  | Process | Process-related effects of bureaucracy, including restrictions, delays, high effort, tasks completed at the expense of substantive work, reduced quality, structure, functionality & quality assurance, and transparency. | - “So it restricts you in your daily work, especially all these clerks, it severely limits them.” (I09) - “When there are certain guidelines you can orient yourself to.” (I12) |
|  | Society | Societal effects of bureaucracy, including delineation, inhibition of innovation, lack of target group orientation, discontinuation of activities, stabilization of society and democracy, legitimization of actions, (control of) enforcement of established regulations. | - “And behind this bureaucratic apparatus, the actual work for the people disappears. For the people in need or those who consult [Association]” (I04) - “It also stabilizes society when there are certain foundations you can orient yourself to” (I12) |
| **Coping strategies and Resources**  [This category includes interview excerpts addressing coping strategies and resources in the bureaucratic work environment, the general professional environment and the private environment.] | Health | Health-related coping strategies and resources, such as exercise, nature, sleep, nutrition, aging processes, vacation planning, personal responsibility, avoidance of unhealthy behavior, preventive medical check-ups, separating. | - “I do a lot of sports. […] It’s my main outlet, so to speak. I always say: 'It’s like psychotropic drugs.'” (I13) - “[…] From that point on, I actually always tried to take control of it myself and actively switch off.” (I09) |
|  | Emotion | Emotional coping strategies and resources, including satisfaction after completing and  enjoyment of substantive work. | - “Yes, it´s always nice when the whole thing bears fruit or when it´s finally done […] Even if it took a long time, at that moment, you think again: ´Yes, it´s finally finished, and now we can get started.”(I09) - “I´m absolutely passionate about my work. I promised my wife that I would never stop working – not at 60, not at 65, not at 70. Because if I´m not allowed to work, I think I´ll get sick. I love working immensely” (I06) |
|  | Cognition | Cognitive coping strategies and resources, such as accepting, time management, awareness of their privileged position, self-efficacy, (self-) reflection, mindfulness, personal traits, guiding principles. | - “There are times when you simply have to let go and say, `I am going to adapt to the circumstances and see what good can come from them” (I04) - “To reflect on situations, to really examine what maybe didn´t go well or why something is bothering me so much” (I05) - “You have to focus on the here and now, on your movements and your sparring partner, because otherwise, things will go wrong” (I08) |
|  | Leadership | Leadership-related coping strategies and resources, such as communication (particularly transparency), coordination, simplify or bypass, trust, controlling results, active managerial role. | - “That´s why my relationship as a leader with the staff is absolutely transparent.” (I12) - “[…] A leader should […] simplify or eliminate things.” (I16) - “I only look at the results now” (I01) |
|  | Inter-personal | Inter-personal coping strategies and resources, such as flexibility and solution-oriented approach of individuals, internal and external support, family, friendships. | - “[…] It only worked because there were people in various positions who said: ´Yes, of course, we are flexible now and will make sure this gets done´” (I04) - “Always seek help – you can´t constantly stew in your own juices.” (I14) |
|  | Process | Process-related coping strategies and resources, including creative leeway, mechanisms of evasion and refusal, internal change initiatives, exploring grey areas, pragmatism, organizational structures. | - “Depends on how much freedom of decision do we actually have within a bureaucratic framework” (I12) - “So you should also have the courage to tackle change” (I14) - “I think at [University 2], we manage quite well to, let´s say, shed light on this gray area” (I10) |
|  | Society | Societal coping strategies and resources, including meaningfulness. | - “[…] Whenever you can come to terms with the purpose behind it, you are also willing to accept inconveniences” (I02) |
